# Fixed Pattern Noise Reduction and Linearity Improvement in Time-Mode CMOS Image Sensors

**DOI:** 10.3390/s20205921

**Published:** 2020-10-20

**Authors:** Miron Kłosowski, Yichuang Sun

**Affiliations:** 1 Faculty of Electronics, Telecommunications and Informatics, Gdańsk University of Technology, 11/12 Gabriela Narutowicza Street, 80-233 Gdańsk, Poland; 2 School of Engineering and Computer Science, University of Hertfordshire, Hatfield, Herts AL10 9AB, UK; y.sun@herts.ac.uk

**Keywords:** image sensor, fixed pattern noise, gain correction, offset correction, integral nonlinearity correction, time-mode ADC

## Abstract

In the paper, a digital clock stopping technique for gain and offset correction in time-mode analog-to-digital converters (ADCs) has been proposed. The technique is dedicated to imagers with massively parallel image acquisition working in the time mode where compensation of dark signal non-uniformity (DSNU) as well as photo-response non-uniformity (PRNU) is critical. Fixed pattern noise (FPN) reduction has been experimentally validated using 128-pixel CMOS imager. The reduction of the PRNU to about 0.5 LSB has been achieved. Linearity improvement technique has also been proposed, which allows for integral nonlinearity (INL) reduction to about 0.5 LSB. Measurements confirm the proposed approach.

## 1. Introduction

Massively parallel image acquisition in CMOS image sensors [1,2,3,4,5] is frequently based on time-to-digit analog-to-digital converters (ADCs) due to a relatively small silicon area and easy pixel integration. Time-mode ADCs are often used due to their advantages such as low noise (fixed pattern and temporal) and high dynamics. An additional benefit is fully parallel analog-to-digital conversion, which makes global shutter implementation easy. In early implementations, ADC integration in the pixels resulted in low fill-factor. Currently, due to the growing popularity of imagers implemented in 3-D technology [6,7,8,9], fill-factor is no more a significant limitation in the application of time-mode converters. Additionally, the separation between the analog and digital domains makes imagers more robust to interference and allows for the use of optimal technology for analog and digital dies. Parallel processing also allows for the implementation of very fast image acquisition (10–1000 kFPS) and preprocessing (if required), which can be used in a machine vision chip [10,11,12,13].

**Figure 1:**
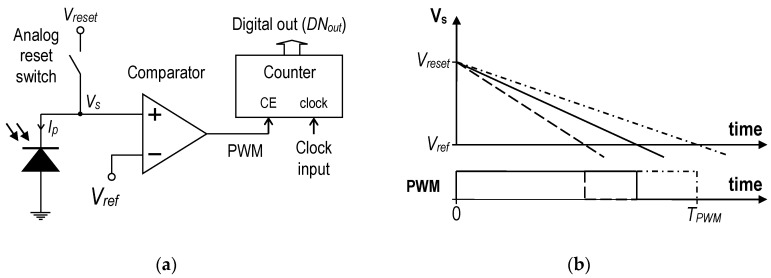
Principle of pixel analog-to-digital converter (ADC) operation in the time mode: (**a**) Simplified schematic diagram; (**b**) Waveforms of the V_s_ and pulse-width modulation (PWM) signals.

Figure 1 shows the operation principle of the time mode-based ADCs. In the beginning, the analog reset switch is closed, which sets the initial voltage V_reset_ on the photodiode. When the analog reset switch is opened, the conversion is started. The photodiode works in the integration mode; therefore, the voltage on the sense-node (V_s_) starts to fall because the sense-node capacitance is discharged with the photocurrent. The analog comparator detects the condition where the V_s_ voltage falls below a certain level (V_ref_) and disables the pulse-width modulation (PWM) signal. This is the end of the conversion. The PWM pulse width and the time of the conversion directly depend on the intensity of the light. The pulse width can be easily converted to a digital value DN_out_ by a time-to-digital converter. A simple binary counter with a clock enable (CE) input (Figure 1a) can be used for this purpose. During the integration, the voltage on the sense-node can be described by the following formula:

dV_s_/dt = −I_p_/(C_j_ + C_s_), (1)

where C_j_ is the junction capacitance of the photodiode, C_s_ is the capacitance of the sense-node, and I_p_ is the photocurrent of the photodiode. The time needed for the voltage V_s_ to reach the reference level V_ref_ (T_PWM_ in Figure 1b) can be easily derived from (1) if C_j_, C_s_, and I_p_ are considered constant during the conversion.

The main aim of the paper is to present new techniques of offset, gain, and linearity corrections in this type of CMOS image sensors with variable V_ref_ voltage. There are solutions in the literature in which the variable V_ref_ voltage is used. In Reference [14], V_ref_ voltage was stepwise changed during the time-to-digital conversion. In References [15,16], a linear ramp of V_ref_ voltage was used in order to obtain a large dynamic range. In Reference [17], noise reduction and dynamic range improvement were achieved by using multiple ramps in the reference voltage. In Reference [18], the hybrid (time and voltage mode) technique of analog-to-digital conversion was introduced, which allows to shorten the conversion time and at the same time maintains the linear light-to-digital characteristic. In the presented paper, an improved technique of the hybrid analog-to-digital conversion with a further reduction of the linearity error and correction of the pixel level gain and offset is proposed. In the following sections, the principle of operation of the proposed hybrid-mode pixel ADC and measurement results are presented and discussed.

## 2. Materials and Methods

**Figure 2:**
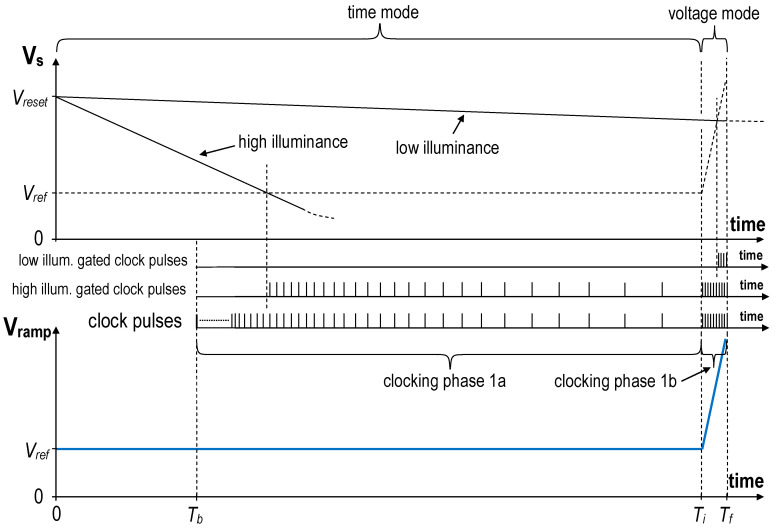
Principle of the hybrid conversion in the proposed pixel-ADC.

Figure 2 shows the principle of the hybrid conversion in the CMOS imager presented in Reference [18]. The conversion starts at the end of the reset pulse (zero on the time axis) and finishes at T_f_. After the blanking time T_b_ (if the photodiode is discharged during this interval the illuminance exceeds the dynamic range of the converter) the clock is enabled and it works until T_f_. In this example, the blanking time T_b_ is 6.6 ms. At the intermediate time moment T_i_, the time mode of operation is changed to the voltage mode.

The voltage mode is only used for low illuminance pixels. When the V_s_ node discharges to the V_ref_ within the time T_i_, then the time mode is used. For the conversion in the time mode (clocking phase 1a) to be linear, the intervals between successive clock pulses are increased [18,19]. It can be easily derived from (1) that without such clock period correction the imager’s digital result of the conversion would be inversely proportional to the photocurrent. In the presented example, the clock period in the clocking phase 1a is 13 µs just after the start of the conversion (T_b_) and 14 ms just before the end of the time-mode clocking phase (T_i_). The blanking phase and the clocking phase 1a take 225 ms in total.

In the voltage mode (clocking phase 1b), the clock frequency is constant and is limited by the delay of the analog comparator. In the presented example, the clock period in the clocking phase 1b is 1.33 µs. There are 15 clock pulses during the clocking phase 1b; therefore, it takes only about 20 µs in total. The influence of the clocking phase 1b and the following offset correction phases (described below) on the total integration time (225 ms) is negligible. The resulting frame rate is around 4 FPS. The system clock frequency is 100 MHz. The time resolution of the time-to-digital conversion is 10 ns.

To maintain good linearity during the entire conversion, the time and voltage modes must be “glued” together in such a way that the following formula is satisfied:

N_v_ = N_t-half_, (2)

where N_v_ is the number of clock pulses in the voltage mode and N_t-half_ is the number of clock pulses in the second half of the time mode interval (T_i_/2 to T_i_). Proof and more details can be found in Reference [18]. Condition (2) must be satisfied to allow a gain correction based on multiplying the response of an individual pixel by a specific coefficient (flat-field correction).

In order to add the functionality of the fixed pattern noise (FPN) (dark signal non-uniformity (DSNU) and photo-response non-uniformity (PRNU)) correction to the presented imager, the modified technique from Reference [20] was applied. The pixel circuit in the presented imager is the same as in Reference [20], but the operating conditions and the controlling waveforms are different.

**Figure 3:**
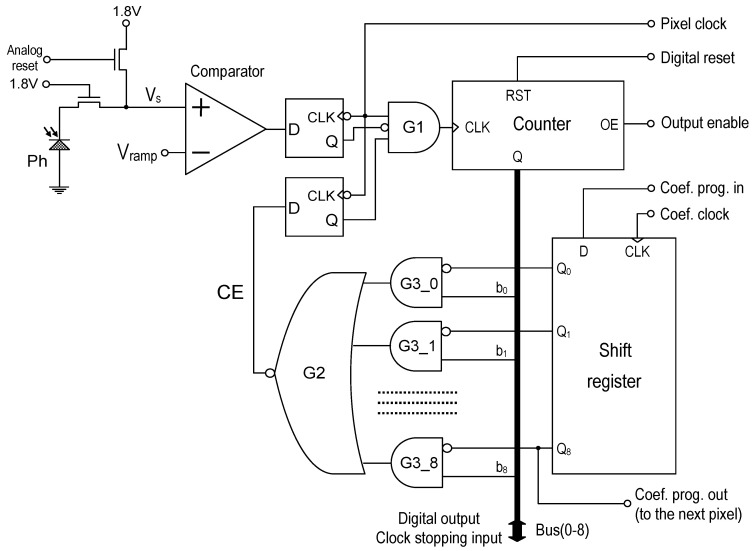
Schematic diagram of the pixel-ADC.

Figure 3 shows a schematic diagram of a pixel ADC implemented in the presented imager. It is equipped with the gain and offset correction circuit from Reference [20], which is based on the clock stopping technique. The G1 gate is used to disable the counter’s clock by the signal from the analog comparator. The counter can also be momentarily stopped by the CE signal generated by the G2 gate. The G3 gates and the coefficient memory (shift register) allow selective blocking of the clock by individual bus lines (b_n_). The bus (common for all pixels) has to be driven by the special pulse train to ensure proper gain and offset correction operation. Since all ADCs operate synchronously, it is possible to use a single bus pulse generator for the entire pixel array.

**Figure 4:**
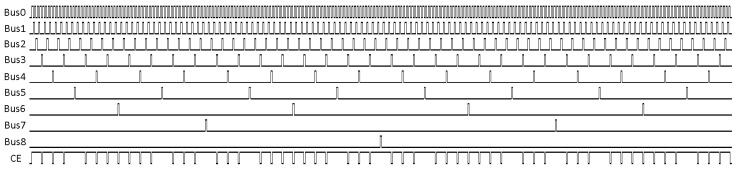
The timing diagram for the Bus(0)–Bus(8) with the gain correction only. The last waveform shows the clock enable (CE) signal (gain correction coefficient: 459/511). For better readability, the constant pixel clock frequency is assumed.

The bus pulse sequence for 9-bit ADCs is presented in Figure 4. The CE signal for pixel with correction coefficient equal to 459/511 (gain correction coefficients can be in the range 0/511–511/511 in the presented clock cycle stealing technique) is also presented. After the conversion, the same bus can be used to transfer data from the pixels (when Output Enable signal coming from the address decoder is activated).

Each pixel has an independent local memory that stores its own correction coefficient for the gain and/or the offset. This memory is implemented by means of a shift register whose serial input is connected to the previous pixel and serial output to the next pixel. This makes the gain correction configuration process straightforward.

Two modifications were introduced to adapt the gain correction mechanism used in a single-slope voltage mode ADC [20] to a time mode operation. The first modification is to take into account that in the time mode the clock is enabled at some point (by the signal from the analog comparator) and it works until the end of the conversion, whereas in the typical single-slope voltage mode, ADC it is enabled at the beginning of the conversion and count until disabled by the signal from the analog comparator. The symmetry of the clock stopping pulse train (Figure 4) on the bus ensures the correct operation of the gain correction in both scenarios. The second modification is that the clock in the time mode should have a variable frequency. The same variable frequency clock is used for the pixel operation and for the clock stopping pulse generator. Therefore, the time-mode conversion works correctly with the gain correction, and the linearity of the conversion is maintained.

**Figure 5:**
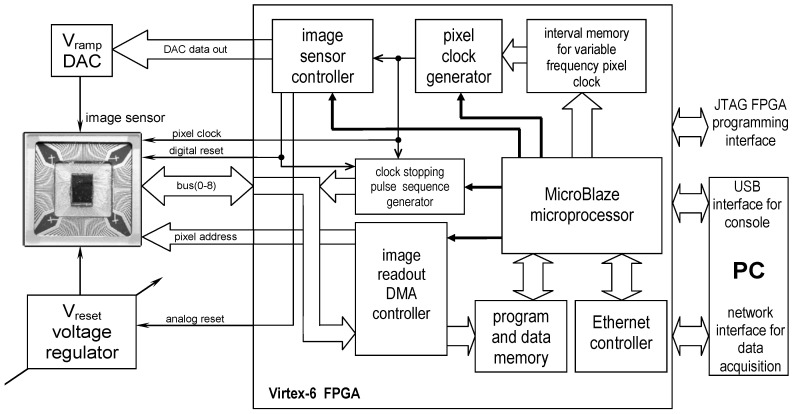
Block diagram of the imager measurement system.

The block diagram of the imager testing and measurement system is presented in Figure 5. It is a modified version of the measurement system presented in Reference [21]. The entire system is controlled by a Xilinx Virtex-6 FPGA and a PC. The module indicated in Figure 5 as the Image Sensor Controller generates the controlling signals for the imager and controls the digital-to-analog converter (DAC) that generates the V_ramp_ signal. The clock stopping pulse sequence generator module implements a special pulse train for gain and offset correction during the conversion (Figure 4). All the control modules are clocked by the pixel clock generator, which can generate a variable frequency clock signal. The successive period values of the pixel clock are read from the interval memory (implemented within FPGA), which can be programmed by the software. There is also a special direct memory access (DMA) image readout controller that implements fast pixel data transfer directly to the system memory. All modules implemented in the FPGA can be configured with the MicroBlaze microprocessor. This microprocessor is implemented in the FPGA with its own on-chip program and data memory. It works under the control of the Linux operating system. The communication with the PC is provided by the Ethernet controller, which enables the transfer of measurement data and access to files on the PC using the Network File System. The Linux system console is connected via the USB interface. The imager measurement results can be processed on a PC using the Octave software, which allows for effective visualization of measurement data. The same PC is used for the FPGA configuration (using the JTAG programming interface) and for the compilation and uploading of the measurement and control software (written in C programming language) for the MicroBlaze processor. Measurements with the variable irradiance were performed using a light source of wavelength 625 nm (LEDs).

**Figure 6:**
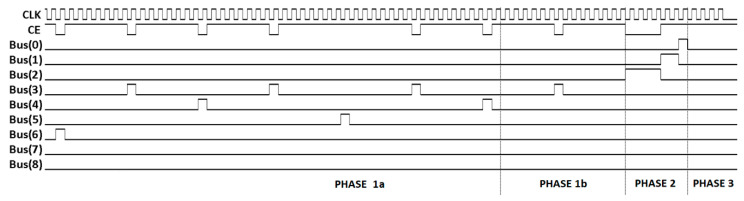
Timing diagram for the Bus(0)–Bus(8) with the gain and offset correction. Only the last part of the waveform has been presented for better clarity. The first waveform shows the pixel clock (for better readability the presented clock frequency is constant in phase 1a). The second waveform shows the pixel clock enable (CE) signal for gain correction coefficient 459/511, offset correction +3, and global offset correction +4.

In Figure 6, the timing diagram of the final part of the conversion is presented. The phase 1a is a time-mode part of the conversion. During this phase, the V_ramp_ voltage is constant and the clock frequency is variable (for better picture clarity the clock in Figure 6 has constant frequency). The phase 1b is a voltage-mode part of the conversion. V_ramp_ voltage is rising and the clock frequency is constant. During the entire phase 1, the gain correction is active (the clock stopping pulses are visible on Bus(3)–Bus(8)). When phase 1 is finished the conversion is ended and all the counters should be enabled by the high level of the V_ramp_ voltage. This allows correct operation of the phase 2, which is intended for offset correction. In the presented example, Bus(0)–Bus(2) lines are used for the clock stopping during the offset correction. Finally, phase 3 is used for the global offset correction (in all pixels because clock stopping is disabled). Phase 3 can be used to reduce the number of bus bits needed for phase 2. In the presented imager, it is possible to change the counting direction globally (reversible counters in the pixels); therefore, phase 3 can be used to reduce the offset to zero when offsets of all pixels are positive.

The bus can be dynamically divided into parts supporting the gain and the offset corrections. In Table 1, the trade-off between the gain and the offset correction range is presented.

## 3. Results

The described technique has been verified using the 128 × 128 pixel imager chip from Reference [20], fabricated in the 0.18 μm 1.8 V CMOS process of ams AG (1P6M). This imager implements the clock stopping circuit in the pixels of the last row of the array (128 pixels). The 128 pixels were tested in the time mode using the described measurement system. The original imager was designed using photo-gate transistors and prepared to operate in a voltage mode. Photo-gate transistors were disabled and parasitic photodiodes Ph were used (implemented by exploiting the exposed drain to substrate junction of an N-channel MOS photo-gate transistor). Parasitic photodiodes are not very sensitive, but it is not so important in presented experiments. Furthermore, the number of 128 pixels is enough to achieve reliable results in statistical measurements of fixed pattern noise (FPN) and integral nonlinearity (INL).

### 3.1. Gain and Offset Correction

Before using the imager, the gain correction coefficients should be obtained. To acquire the gain correction coefficients, the imager should be uniformly illuminated. Then, the imager’s response should be measured. The coefficients are calculated with respect to the weakest pixel response for which the coefficient is equal to one. Other coefficients should be calculated to attenuate the pixel responses to the level of the weakest pixel response. Offset correction data is acquired by measuring the imager’s response in the dark condition. Then, for each pixel, correction data is calculated, which will be added to the pixel response in phase 2. The offset correction data can now be combined with the gain correction coefficients and transferred to the imager’s coefficient memory. After that phase, flat-field correction is achieved, but all pixels have non-zero dark value. In phase 3, responses of all pixels can be shifted down to the zero value. In the presented example, linear-feedback shift register (LFSR) counter is used and valid states are in the range 1–511. Zero is an invalid state and value one represents a dark condition instead. It is also a good idea to shift the counters in phase 3 to the value even higher than the value representing the dark condition (value one in this example). The counters in the presented imager do not stop on reaching the lowest value, they roll over to the highest value instead. Therefore, random noise can make the pixels flicker between 1 and 511. In the following measurements, value 2 was used as the offset correction target and no flickering has been observed.

**Figure 7:**
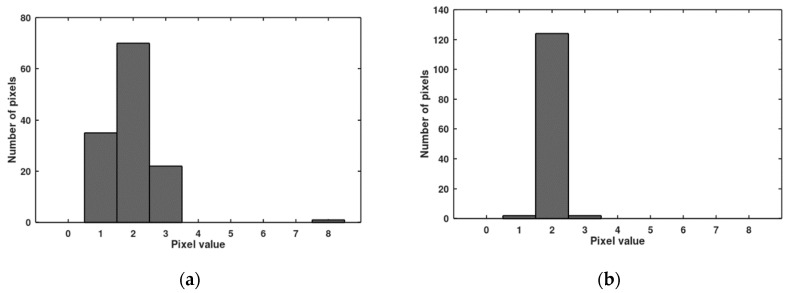
Distribution of dark signal responses before (**a**) and after (**b**) the offset correction for the measured imager (containing 128 pixels).

**Figure 8:**
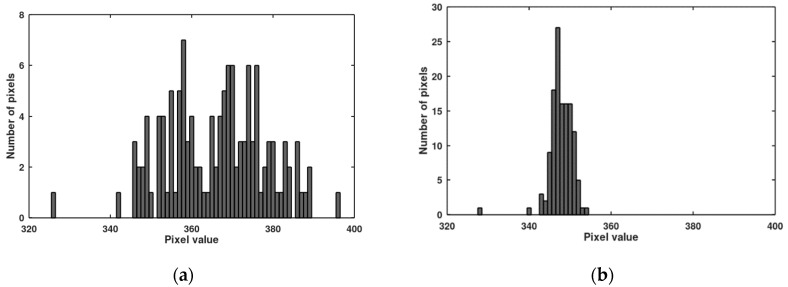
Distribution of photo signal responses before (**a**) and after (**b**) the gain correction for the measured imager. The offset correction takes precedence over the gain correction.

**Figure 9:**
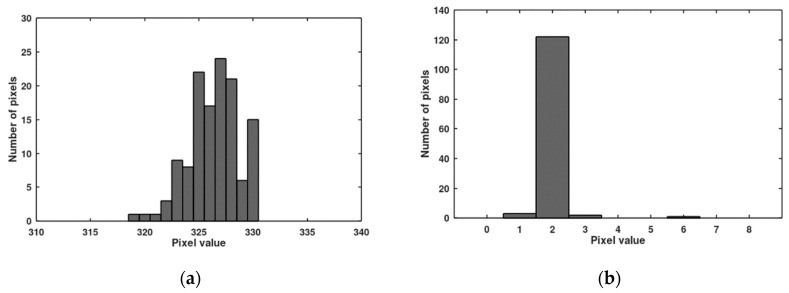
Distribution of photo signal responses (**a**) and dark signal responses (**b**) when the gain correction takes precedence over the offset correction.

In Figure 7, histograms of pixel responses before and after the offset correction are presented (value 2 is used as the target of the offset correction). Because of one outstanding pixel (value 8), it was necessary to use 3 bits of the bus for the offset correction. In Figure 8, histograms of pixel responses before and after the gain correction are presented. The correction is not full because only 6 lines of the bus were left for the gain correction (precedence of the offset correction). Seven lines of the bus are enough to cover the gain non-uniformity, but then only 2 bits are left for the offset correction (precedence of the gain correction). Histograms of pixel responses for this situation are shown in Figure 9.

**Figure 10:**
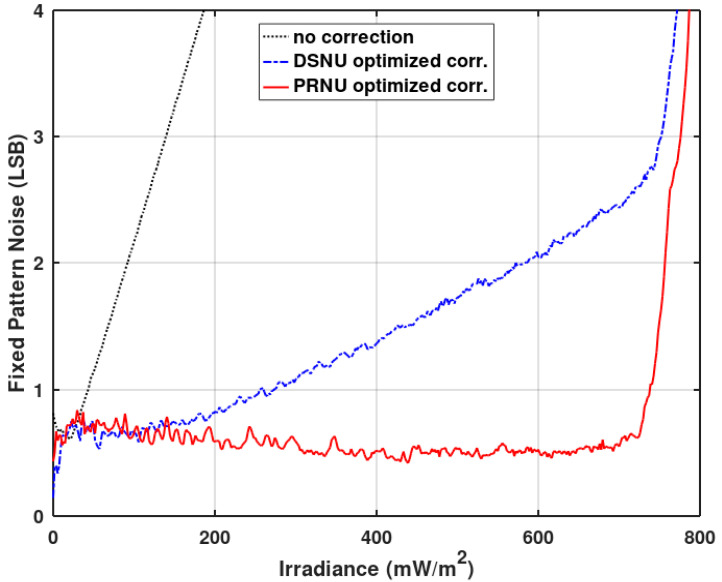
Measured fixed pattern noise (FPN) of the presented imager as a function of the irradiance without corrections, with the offset and gain correction (offset correction takes precedence), and with the offset and gain correction (gain correction takes precedence).

**Figure 11:**
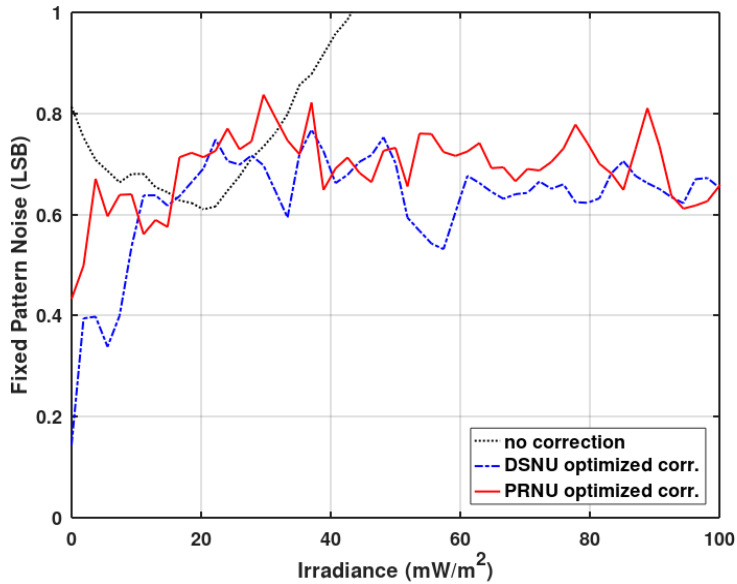
Measured low irradiance FPN region of the presented imager as a function of the irradiance without corrections, with the offset and gain correction (offset correction takes precedence), and with the offset and gain correction (gain correction takes precedence).

The trade-off between gain and offset correction can be also seen in Figure 10, where the fixed pattern noise of the imager in the function of the irradiance is presented. The PRNU (the light component of FPN) has been substantially reduced. It is also evident that the gain correction usually should take precedence over the offset correction for time mode-based imagers. The DSNU (the dark component of FPN) reduction achieved by the offset correction is only visible for very low irradiances. For better clarity, Figure 11 shows the FPN for low irradiances. Figure 10 also shows an increase in the PRNU for high irradiances. Without the gain correction, all pixels reach the same maximum value (at saturation). With the gain correction enabled, the saturation will cause the PRNU to reappear (the same saturation value multiplied by a different correction coefficient shows up as a fixed pattern noise). This can be resolved by enforcing the same upper limit in all pixel counters.

Due to the possibility of dynamically changing the clock stopping bus partitioning, it is possible to automatically tune the trade-off of the corrections depending on the scene brightness. For dark scenes, more bus lines should be assigned to the offset correction and for bright scenes to the gain correction.

### 3.2. Linearity Improvement

**Figure 12:**
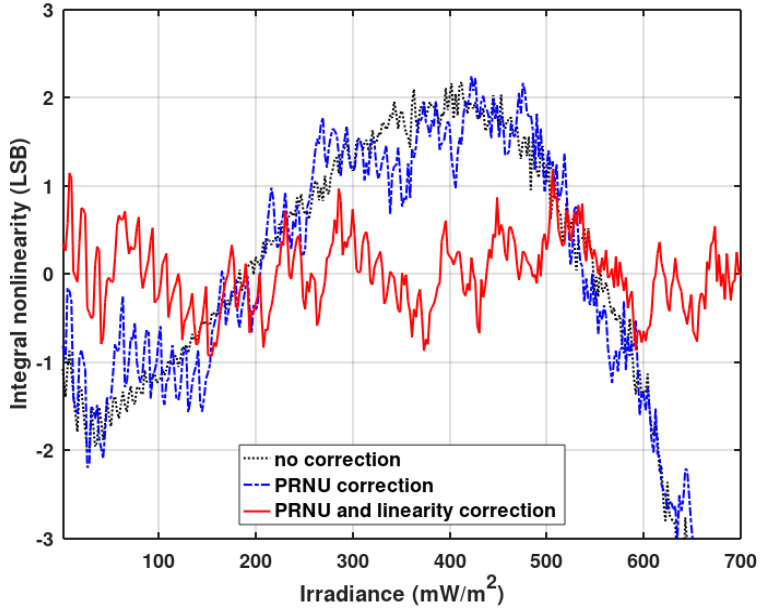
Integral nonlinearity (INL) measured for a single randomly chosen pixel as a function of the irradiance with no corrections, with the photo-response non-uniformity (PRNU) (gain) correction, and with the PRNU and linearity correction.

Integral nonlinearity (INL) can be degraded due to the clock stopping in the gain-corrected pixel. The unevenness of the stopped clock depends on the gain correction coefficient and the pixel output value. In Figure 12, the INL measured for the single pixel in the function of the irradiance is presented. Single-pixel measurement is obligatory because pixels have different photo-responses and the pattern of the irregularity depends on the response of the pixel and the gain correction coefficients. Therefore, the irregularity pattern will not be well visible when many pixel responses are averaged.

Table 2 shows the maximum possible INL values calculated by simulation for various resolutions of the pixel ADCs. It is worth noting that although the max. INL error grows slowly with the increasing resolution, the same INL error expressed as a percentage of the full-scale decreases with the increasing resolution of the ADC. Thus, the more accurate the pixel’s ADC, the smaller the INL error introduced by the presented gain correction technique.

As seen in Figure 12, the gain correction introduces irregular deviations to the INL measured without the correction. Nevertheless, the maximum INL without the correction is still higher than those irregular deviations. Thus, it is worth making some improvements to reduce the nonlinearity of the analog path of the ADC, at least to the level of gain correction induced nonlinearity. The linearity optimization procedure is described below. The result of this procedure is presented in Figure 12 (red graph—PRNU and linearity correction).

Linearity correction is possible due to the ability to fine-tune each clock period during time-mode analog-to-digital conversion. By default, they are theoretically calculated from (1) and stored in the interval memory of the pixel clock generator. Then, it is possible to correct the interval memory contents to obtain better linearity. Obtaining correction values from theoretical formulas is difficult due to the large number of secondary effects affecting linearity. The authors propose a rudimentary optimization procedure that allows to obtain satisfactory results after several iterations.

First, the INL nonlinearity is measured, then it is converted into DNL nonlinearity (by means of numerical differentiation). Then, the resulting DNL is smoothed numerically. The DNL is interpolated to determine its values corresponding to the consecutive digital numbers of the imager’s response. For each imager’s digital response, a clock period correction proportional to the DNL error value is determined. This correction value is multiplied by a specific ‘eta’ coefficient selected experimentally, which affects the speed of optimization.

Finally, the imager’s integration time has to be corrected. The sum of all clock periods after the optimization step differs from the initial one, which cause the change of the integration time. This is corrected in the last step. The Algorithm 1 described in the Octave language is given below.
**Algorithm 1.** The Octave language code of the proposed linearity improvement algorithm1: % **Input:** reci (vector containing current clock periods for time-mode ADC)2: % **Input:** inl (vector containing current result of INL measurement)3: % **Input:** samples (vector containing photo-response of the imager used for INL measurement)4: eta = 0.5; % correction coefficient5: rng = find(inl);6: numdiff = diff(inl)’; % DNL approximation7: numdiff = [0; numdiff];8: [yh, lambda] = regdatasmooth(rng, numdiff); % DNL smoothing9: diff_inter = interp1(samples, yh, 16:510); % DNL interpolation10: % 16 – 510 are the digital response range for the time-mode phase of the conversion11: sum_reci = sum(reci); % total integration time (original)12: reci += round(reci .* (diff_inter .* eta)’); % correction of the clock periods13: sum_corr = sum(reci); % total integration time (distorted after INL correction)14: final_corr = sum_corr / sum_reci; % calculation of the integration time correction coefficient15: reci = round(reci ./ final_corr); % final correction of the clock periods16:% **Output:** reci (vector containing corrected clock periods for time-mode ADC)

**Figure 13:**
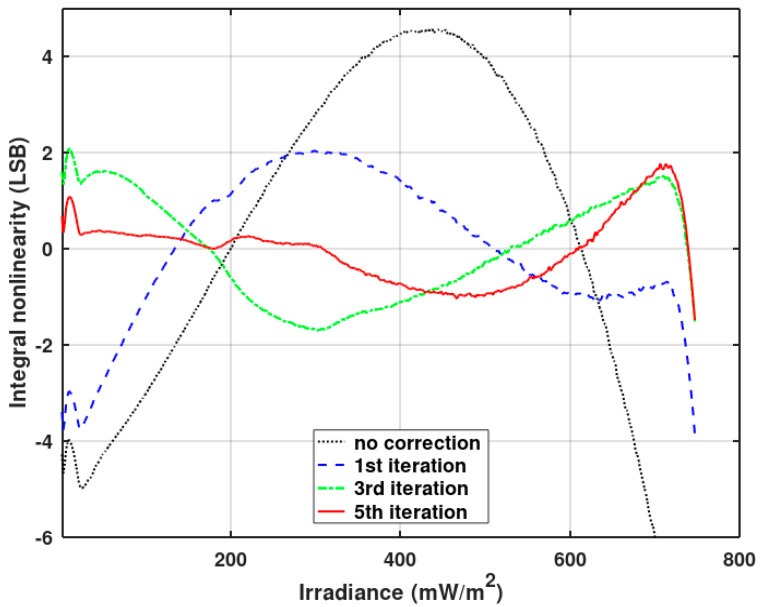
The results of the initial iterations of the linearity correction (with the gain and offset correction disabled). The measured INL is an average of 128 pixels.

Figure 13 shows the results of the first iterations of the presented algorithm. There is a significant reduction of the integral nonlinearity.

**Figure 14:**
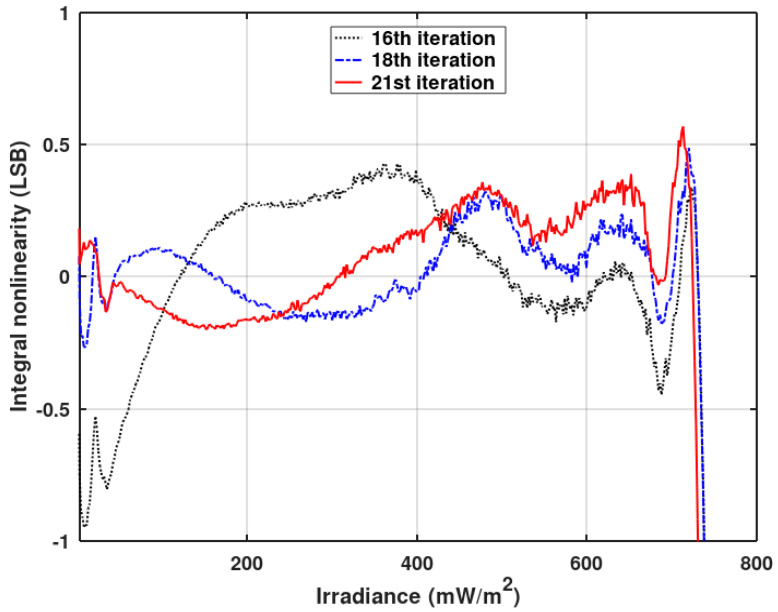
The results of the final iterations of the linearity correction (with the gain and offset correction disabled). The measured INL is an average of 128 pixels.

**Figure 15:**
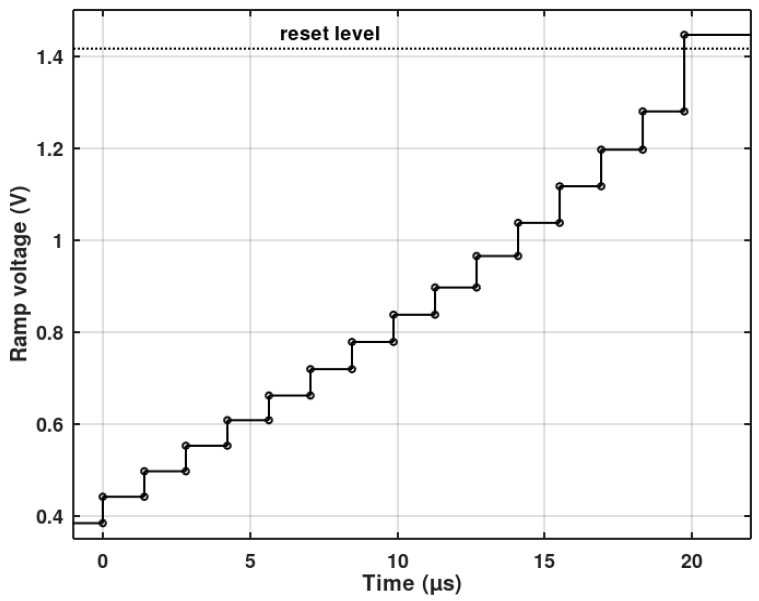
Ramp signal after linearity optimization of the voltage-mode phase 1b of the proposed pixel-ADC. The last ramp step ensures that all pixel counters are turned on before the start of the offset correction phase.

The following iterations were performed after reducing the ‘eta’ coefficient to 0.2. The results are shown in Figure 14. Both figures show a linearity distortion for low irradiation levels. This is due to pixel ADCs going into voltage mode, which is not included in the presented linearity optimization algorithm. The final 21st iteration was performed after optimization of the waveform of V_ramp_ voltage, the principles of which are presented in Reference [18]. Figure 14 shows the improvement of the INL waveform for the low illumination level after the V_ramp_ optimization (red plot) based on a semi-stochastic algorithm [22]. Figure 15 shows the waveform of the V_ramp_ after the aforementioned optimization. The visible nonlinearity of the ramp is associated with the linearity optimization performed for the voltage mode (phase 1b). The last V_ramp_ step had to be modified—it has to reach a voltage above the pixel reset voltage level. This is needed to ensure that all pixel counters (even the completely dark ones) are activated to allow the offset correction (phase 2 and 3).

**Figure 16:**
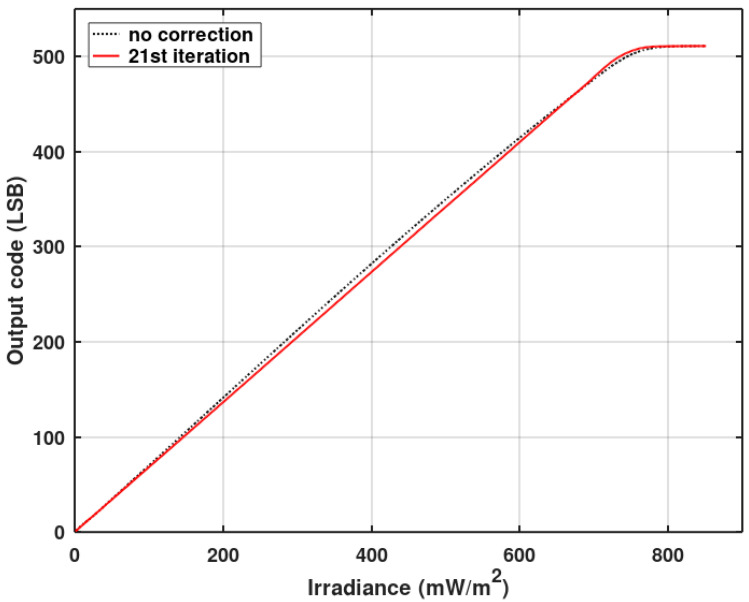
The measured response of the imager (with the gain and offset correction disabled). The presented response is an average of 128 pixels.

Figure 13 and Figure 14 show INL averaged over 128 pixels operating without a gain correction. Figure 12 also shows the improvement of INL for pixels working with a gain correction (red plot) after the described pixel clock correction. A slight influence of the gain correction on INL is still visible. Offset correction has no effect on INL. Figure 16 shows the imager’s response (digital output code) vs. irradiance for the full dynamic range. The black plot presents the original imager’s response before any optimizations, and the red plot depicts the response after the final INL optimization iteration. The linearity improvement is clearly visible.

## 4. Discussion and Conclusions

The paper presents the technique of clock stopping-based gain and offset correction applied to time-mode ADCs implemented in pixels of a CMOS imager chip. The technique was confirmed with measurements demonstrating a significant reduction of FPN noise. A reduction of FPN to 0.5 LSB was achieved. The flexibility of the solution allows a trade-off between gain correction and offset correction precisions. A similar technique has previously been used for voltage-mode ADCs. In this paper, it has been modified for time-mode and hybrid time and voltage mode ADCs.

The linearity of converters using the gain correction is slightly degraded, but relative INL is limited to the acceptable level and decreases with the increase of the resolution of the pixel ADCs. The authors also propose a simple method for improving the linearity of the pixel ADC working in the time-mode. Thanks to the proposed method, it was possible to reduce the INL to about 0.5 LSB (from the initial 4 LSB).

Gain correction effectively reduces FPN and not only improves image quality but also protects against using FPN of images produced by the imager as its fingerprint [23]. The PRNU correction implemented early in the image processing flow (directly in the pixel ADC) makes the imager very secure, because it is difficult to bypass the PRNU correction and the rest of the image processing algorithms operate on the flat field corrected picture, so they will not add PRN-based artifacts.

The implemented pixel occupies an area of 20 × 36 μm, while the photosensitive area is only 5 × 5 μm. Due to the increasing popularity of vertically stacked imagers with capacitive [24] and galvanic [25,26] coupling, the fill-factor of time-mode and hybrid-mode pixels can be improved by moving the gain correction circuitry entirely to the digital layer. In the stacked architecture, the technology used for the digital parallel processing layer of the stack can be chosen that the area of this layer is similar to the area of the photosensitive analog layer and, therefore, it is possible to match the layers. In the future, it will be probably possible to add more layers to the stacked architecture.

## Figures and Tables

**Table 1:** The trade-off between the gain and the offset correction range for the presented imager.

Number of Bits for Gain Correction	Programmable Range of Gain Correction Coefficient	Programmable Range of Offset Correction	Number of Bits for Offset Correction
9	0–1	0–0	0
8	0.501–1	0–1	1
7	0.751–1	0–3	2
6	0.877–1	0–7	3
5	0.939–1	0–15	4
4	0.971–1	0–31	5
3	0.986–1	0–63	6
2	0.994–1	0–127	7
1	0.998–1	0–255	8
0	1–1	0–511	9

**Table 2:** Max. INL in LSBs and in the percentage of the full-scale (for all output codes and gain correction coefficients) simulated as a function of the ADC resolution.

ADC Resolution (bits)	Max. INL (LSB)	Max. INL (%)
6	1.000000	1.58730
7	1.110236	0.87420
8	1.333333	0.52287
9	1.444227	0.28262
10	1.666667	0.16291
11	1.777724	0.08684
12	2.000000	0.04884
13	2.111098	0.02577
14	2.333333	0.01424
15	2.444441	0.00746
16	2.666667	0.00406
17	2.777777	0.00211
18	3.000000	0.00114
